# Treatment of Obesity

**Published:** 1891-03

**Authors:** 


### Treatment of Obesity.

In an article on the "Physiological Treatment of Obesity" in the N. Y. *Medical Record*, February 15th, 1890, Dr. Walter Mendelson gives the following diet list made up as an average of two, by Oertel, and somewhat modified for American habits. Such a list is only to serve as a *general guide* to the patient to whom it is to be given. No absolutely hard-and-fast rules can be laid down, and patients under treatment should be seen—and weighed—from time to time; increasing one kind of food and diminishing another as occasion demands.

*Breakfast:* 1 cup (6 ounces) tea or coffee, with milk and sugar; Bread, $2\frac{1}{2}$ ounces (2 or 3 slices); butter, $\frac{1}{2}$ ounce; 1 egg or $1\frac{1}{2}$ ounces meat.

*Dinner:* Meat or fish, 7 ounces; green vegetables, 2 ounces (spinach, cabbage, string beans, asparagus, tomatoes, beet tops, etc.); farinaceous dishes, $3\frac{1}{2}$ ounces (potatoes, rice, hominy, macaroni, etc.), or these may be omitted and a corresponding amount of green vegetables substituted; salad, with plain dressing, 1 ounce; fruit, $3\frac{1}{2}$ ounces; water, sparingly.

*Supper or Lunch:* 2 eggs, or lean meat 5 ounces; salad (radishes, pickles, etc.), $\frac{3}{4}$ ounce; bread, $\frac{3}{4}$ ounce (1 slice); fruit, $3\frac{1}{2}$ ounces, or fruit may be omitted and bread, 2 ounces, substituted; fluids (tea, coffee, etc.), 8 ounces. No beer, ale, cider, champagne, sweet wines, and spirits. Claret and hock in great moderation. Milk, except as an addition to tea or coffee, only occasionly. Eat no rich gravies, and nothing fried. Patients should always feel *better*—never worse—under treatment. Lassitude and fatigue are signs that the muscular tissue, as well as the fat, is being reduced, and that more non-nitrogenous food must then be allowed.

Dr. Mendelson says: Never yield to the wishes of the patient to grow thin *quickly*. All reforms, to be lasting and beneficial, must be slow in action; they must be the result of education; they must be a growth from within, not an impress from without. And the cells of the body, in their infinite diversity of occupation resembling the citizens of a state, can by slow degrees be habituated to better things, to change their vicious mode of action to one harmonious with the welfare of the commonwealth. And when this education has once been established, continuance becomes a mere habit.—*Annals of Hygiene.*

